# Supplementary material for: Eomesodermin in conjunction with the BAF complex promotes expansion and invasion of the trophectoderm lineage
Source: Nat Commun. 2025 May 31;16:5079. doi: 10.1038/s41467-025-60417-w (PMC12126495; doi:10.1038/s41467-025-60417-w)
Supplement: Supplementary file 1 — Supplementary Information [file 41467_2025_60417_MOESM1_ESM.pdf]

Supplementary Information

Eomesodermin in conjunction with the BAF complex promotes expansion and invasion of the trophectoderm lineage

Alexandra Maria Bisia, Maria-Eleni Xypolita, Elizabeth K. Bikoff, Elizabeth J. Robertson, Ita Costello

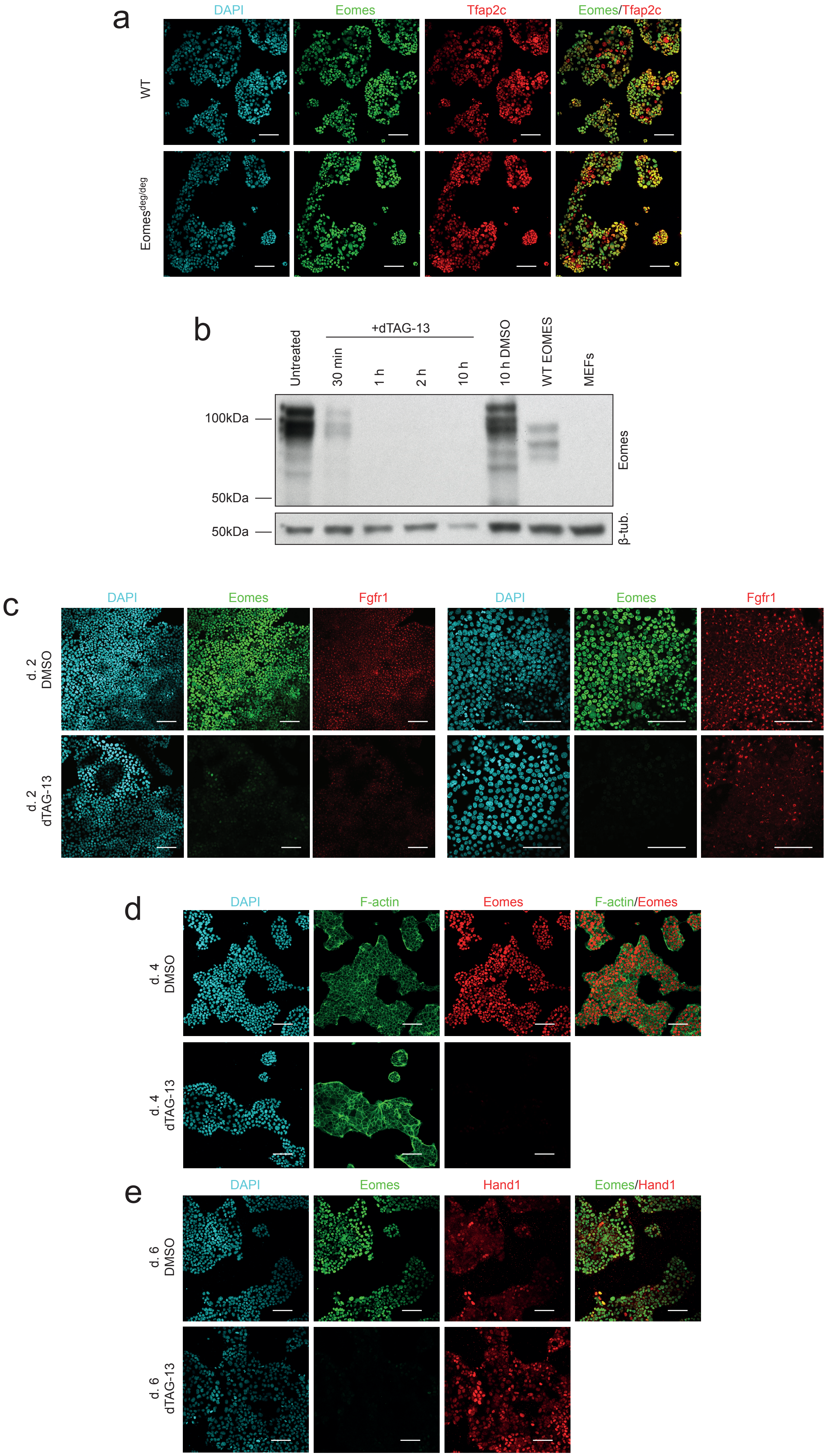
a
DAPI
Eomes
Tfap2c
Eomes/Tfap2c
WT
Eomes deg/deg
b
Untreated
+dTAG-13
30 min
1 h
2 h
10 h
10 h DMSO
WT EOMES
MEFs
100kDa
50kDa
Eomes
50kDa
β-tub.
c
DAPI
Eomes
Fgfr1
d. 2 DMSO
d. 2 dTAG-13
d
DAPI
F-actin
Eomes
F-actin/Eomes
d. 4 DMSO
d. 4 dTAG-13
e
DAPI
Eomes
Hand1
Eomes/Hand1
d. 6 DMSO
d. 6 dTAG-13

Supplementary Figure S1: **TSCs downregulate stemness and upregulate differentiation markers upon Eomes depletion**

(a) Immunofluorescence staining of wild-type and Eomes$^{deg/deg}$ TSCs for Eomes and Tfap2c expression. Scale bar: 100 um.

(b) Immunoblot analysis of Eomes degradation timecourse in dTAG-13-treated Eomes$^{deg/deg}$ TSCs. Mouse embryonic fibroblasts were included as a negative control.

(c-e) Immunofluorescence staining of Eomes-depleted and control DMSO-treated Eomes$^{deg/deg}$ TSCs analysed for expression of TSC marker Fgfr1 (c), F-actin (phalloidin) (d), or early TB differentiation marker Hand1 (e), alongside Eomes. Scale bar: 100 um.

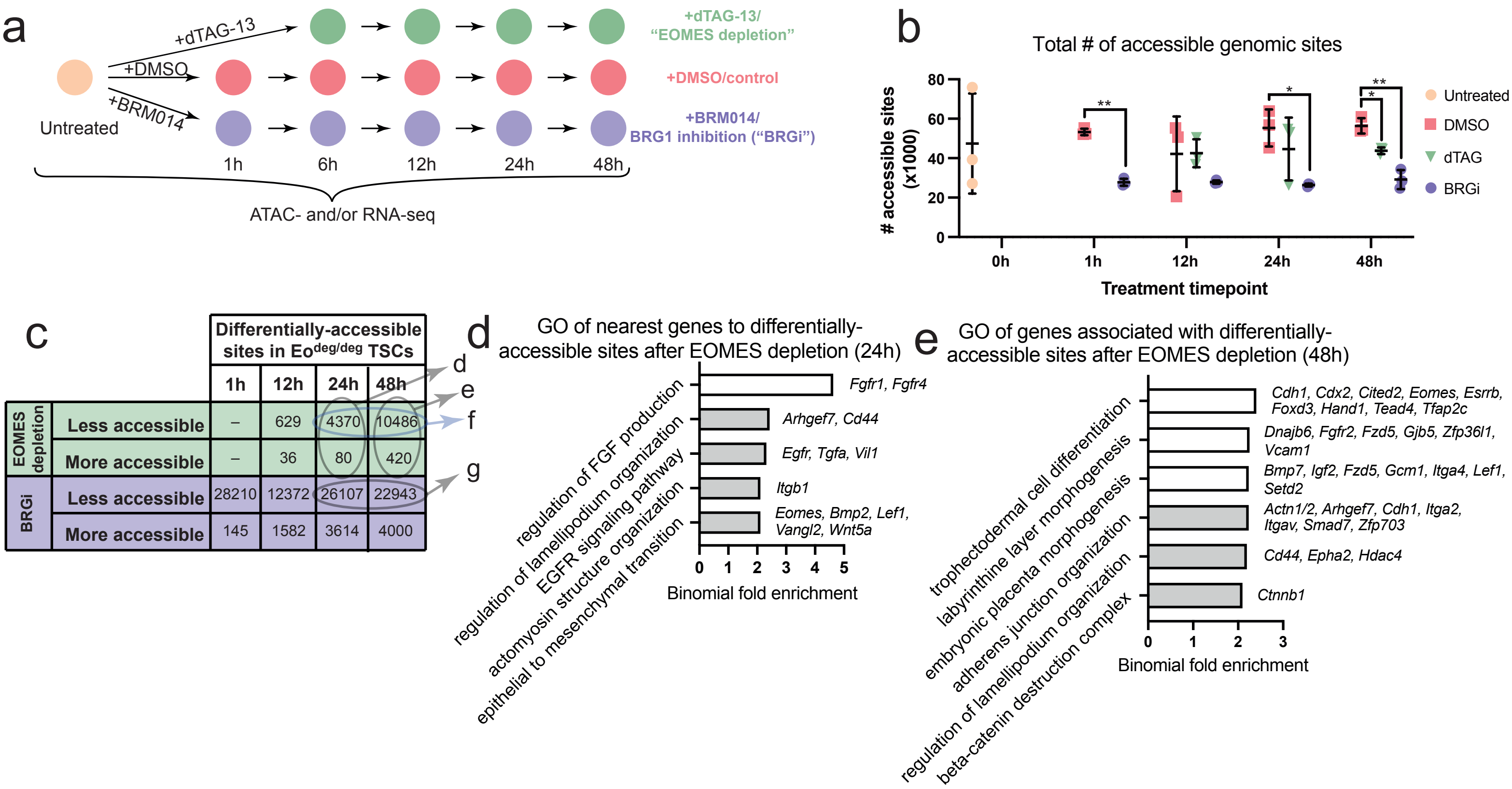

f

Enriched motifs in reduced accessibility sites upon EOMES depletion

Factor name	EOMES interactor	24h Target seqs with motif (%)	24h P-value	48h Target seqs with motif (%)	48h P-value	Motif
TFAP2C	Y	19.8	1e-136	25.5	1e-430	
JUNB	Y	15.5	1e-142	17.0	1e-469	
KLF5	Y	17.1	1e-38	19.4	1e-75	
ELF5	Y*	12.5	1e-26	13.1	1e-81	
ESRRB	Y	9.5	1e-5	9.0	1e-8	
POU3F1 (OCT6)	Y	4.4	1e-3	4.5	1e-13	
PBX3	Y	3.2	1e-2	3.1	1e-6	
ZFP281	Y	–	–	2.9	1e-3	
SOX21	N	38.3	1e-174	38.2	1e-466	
SOX15	N	24.6	1e-148	26.1	1e-441	
SOX2	N	24.0	1e-189	24.6	1e-486	
TEAD4	N	18.3	1e-96	17.9	1e-211	
MEIS1	N	29.1	1e-44	23.7	1e-27	
CDX2	N	9.4	1e-20	8.6	1e-41	
RBPJ	N	–	–	16.2	1e-4	
EOMES	–	56.3	1e-473	41.7	1e-462	

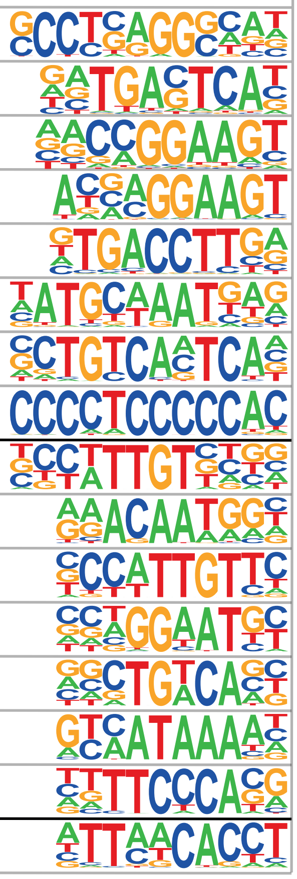

g

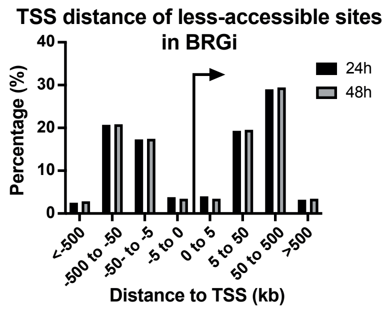

Supplementary Figure S2: Experimental protocol for ATAC- and RNA-seq of Eomes-depleted or Brg1-inhibited TSCs

(a) Schematic of time-course experimental setup showing Eomes depletion and BRGi conditions, as well as timepoint-specific DMSO controls. Each experimental condition was carried out in triplicate.

(b) Summary statistics of total accessible site numbers in each experimental condition outlined in (a). Produced using a two-sided t-test, *: p=,0.05; **: p=,0.01.

(c) Summary statistics of differentially-accessible peaks in each experimental condition outlined in (a).

(d-e) Selected enriched GO terms associated with the genes nearest to differentially-accessible sites after 24 (d) or 48h (e) of BRGi. White bars correspond to TE-related terms, grey bars correspond to cell interactions and motility-related terms.

(f) Selected enriched consensus motifs at sites of reduced accessibility after 24 and 48h of Eomes depletion.

(g) Bar graph of distance distribution of sites of reduced accessibility from the nearest TSS after 24 and 48h of BRGi.

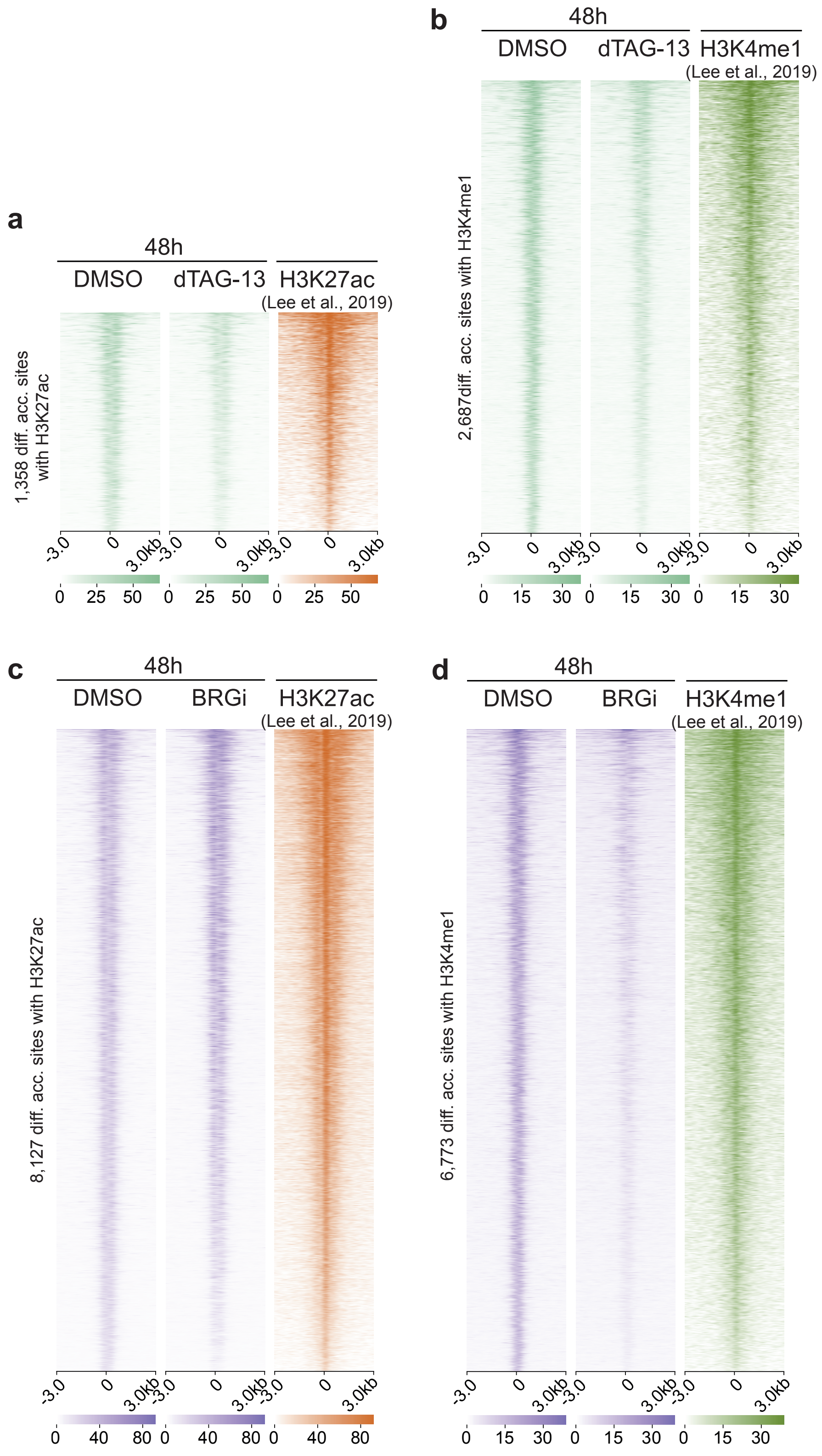

e

		Differentially-accessible sites (#)	H3K27ac (%)	H3K4me1 (%)	Total (%)
**Eomes depletion**	**12h**	664	2.3	17.8	19.0
	**24h**	4,449	4.6	19.5	22.3
	**48h**	10,905	12.5	24.6	33.6
**BRGi**	**6h**	2,658	67.0	10.3	75.2
	**12h**	13,953	25.4	25.7	46.6
	**24h**	29,720	29.7	25.2	50.3
	**48h**	26,942	30.2	25.1	50.7

Supplementary Figure S3: Eomes depletion- and BRGi-induced differentially-accessible sites coincide with active and poised enhancer marks.

(a) Heatmap of the overlap between Eomes depletion-induced differentially-accessible sites and previously-published H3K27ac occupancy in TSCs (GEO acc. code GSM3019290).

(b) Heatmap of the overlap between Eomes depletion-induced differentially-accessible sites and previously-published H3K4me1 occupancy in TSCs (GEO acc. code GSM3019292).

(c) Heatmap of the overlap between BRGi-induced differentially-accessible sites and previously-published H3K27ac occupancy in TSCs [GEO acc. code as in (a)].

(d) Heatmap of the overlap between BRGi-induced differentially-accessible sites and previously-published H3K27ac occupancy in TSCs [GEO acc. code as in (b)].

(e) Summary statistics of overlap between differentially-accessible sites and previously-published H3K27ac or H3K4me1 occupancy in TSCs at different timepoints of Eomes depletion or BRGi.

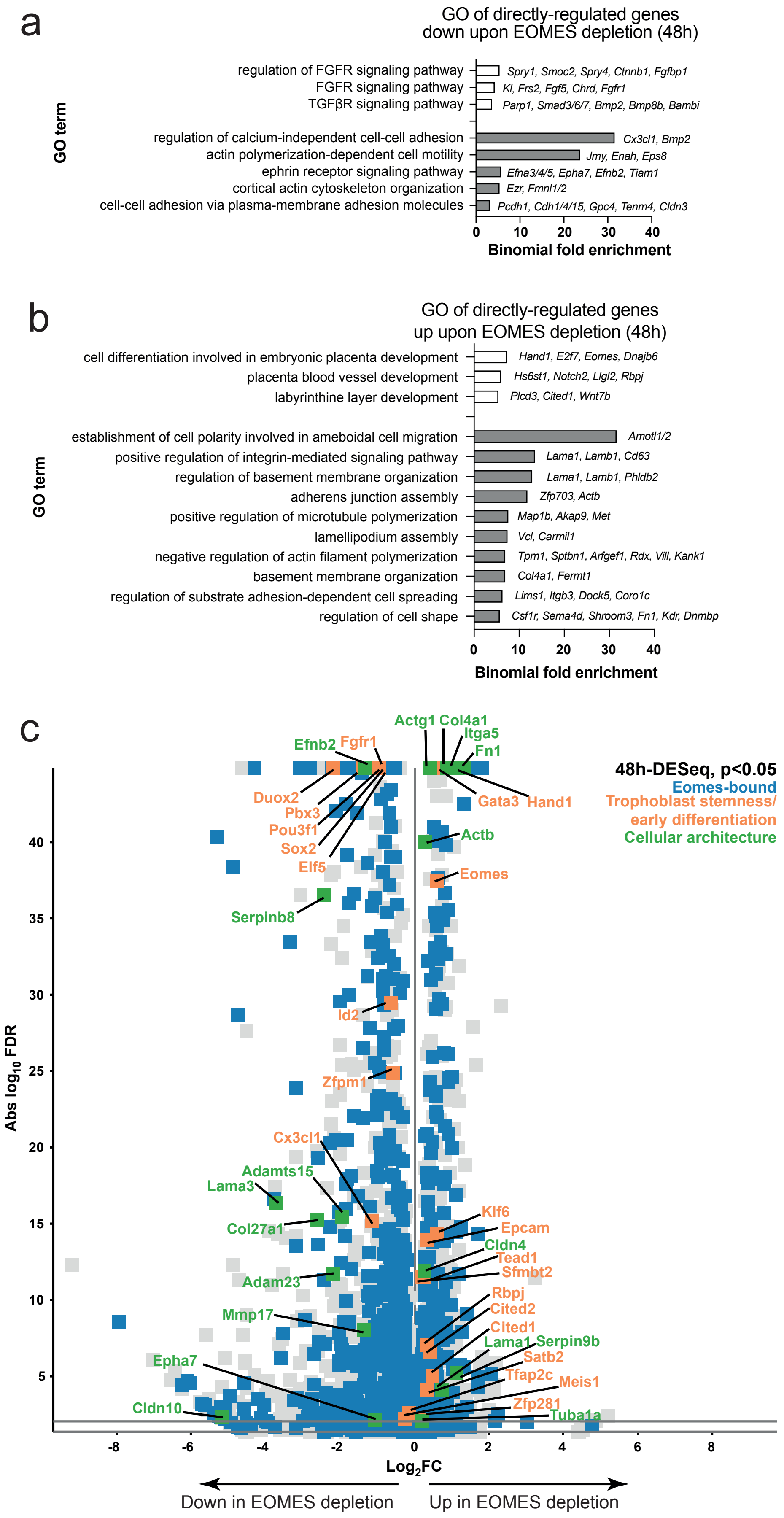

Supplementary Figure S4: Eomes depletion results in misregulation of TSC-related genes.
(a-b) GO terms enriched in genes downregulated (a) or upregulated (b) in Eomes-depleted TSCs after 48h of dTAG-13 addition
(c) Volcano plot of differentially-expressed genes between Eomes-depleted and control TSCs 48h after dTAG-13 addition. Trophoblast stemness/early differentiation (red) and cellular architecture (green) genes are also a subset of Eomes-bound genes (blue)

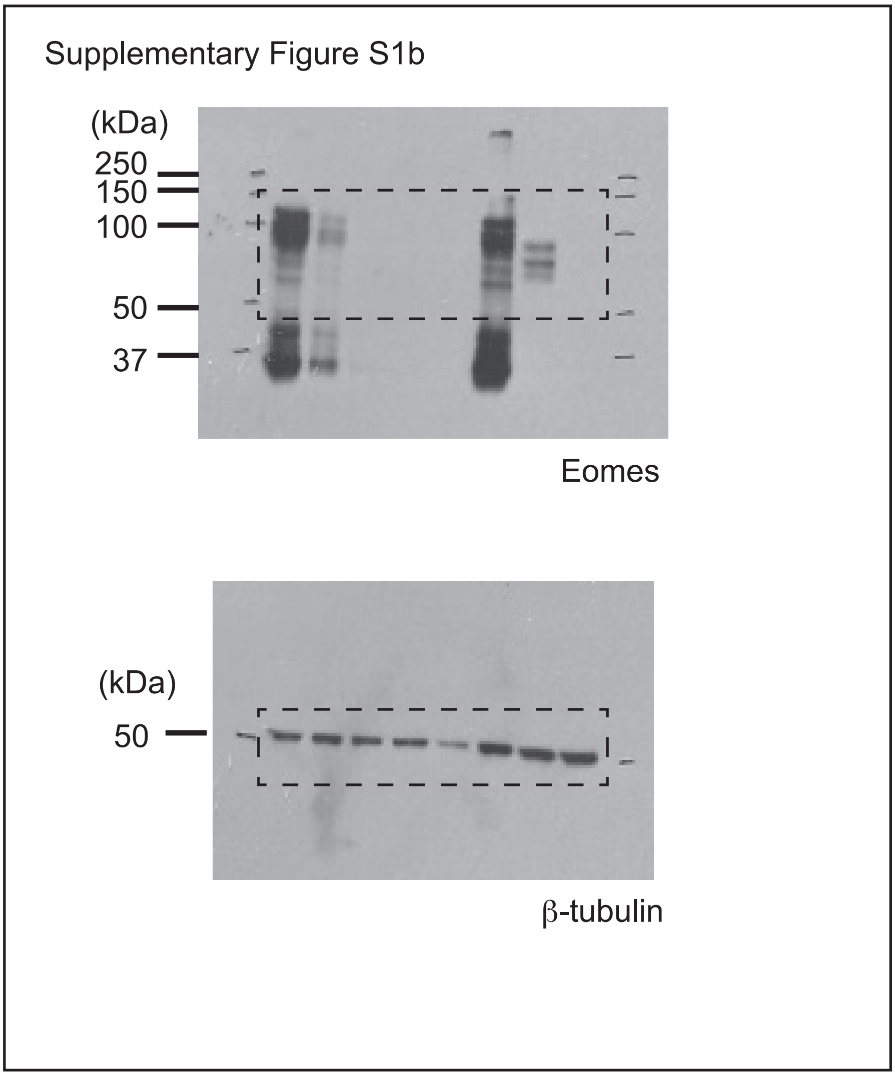

Supplementary Figure S5: Full size images of Western blots from Supplementary Figure S1

Supplemental Table 1: *Antibodies used in this study*

Reagent	Source	Identifier	Lot #	RRID	Dilution	Application
Rat monoclonal IgG2a anti-Eomes	eBioscience	14-4875-82	2493129	AB_11042577	1:500	Immunofluorescence (IF)
Rabbit polyclonal anti-AP-2γ	Cell Signaling	2320S	3	AB_2202287	1:100	IF
Rabbit monoclonal IgG anti-FGFR1	Cell Signaling	9740S	4	AB_11178519	1:100	IF
Phalloidin AF633	ThermoFisher	A22284			1:100	IF
Polyclonal goat anti-HAND1	R&D	AF3168	WTD0423011	AB_2115853	1:100	IF
Rabbit polyclonal anti-RFP	Rockland	600-401-379	48710	AB_2209751	1:200	IF
Donkey anti-rat AF488	Invitrogen	A-21208	2482958	AB_2535794	1:400	IF
Donkey anti-rat AF594	Invitrogen	A-21209	2078918	AB_2535795	1:400	IF
Donkey anti-rabbit AF488	Invitrogen	A-21206	1927937	AB_2535792	1:400	IF
Donkey anti-goat AF594	Invitrogen	A-11058	2445414	AB_2534105	1:400	IF
Donkey anti-rabbit AF594	Invitrogen	A-21207	2313074	AB_141637	1:400	IF
Rat monoclonal IgG2a anti-Eomes	eBioscience	14-4875-82	2493129	AB_11042577	1:10,000	Western Blot (WB)
Rabbit polyclonal anti-β-tubulin	Cell Signaling	2146S	10	AB_2210545	1:2,000	WB
Goat anti-rat IgG, HRP conjugated	Cell Signaling	7077S	14	AB_10694715	1:2,000	WB
Donkey anti-rabbit IgG, HRP conjugated	Amersham	NA934V	17271476	AB_772206	1:2,000	WB
Guinea Pig anti-Rabbit IgG (Heavy & Light Chain)	Antibodies online	ABIN101961	NE-200-022001	AB_10775589	1:100	CUT&RUN
Rabbit polyclonal anti-Eomes	abcam	ab23345	GR3304549-1	AB_778267	1:50	CUT&RUN
Rabbit polyclonal anti-Eomes	abcam	ab23345	GR3390346-2	AB_778267		RIME
