# Supplementary material for: Eomesodermin in conjunction with the BAF complex promotes expansion and invasion of the trophectoderm lineage
Source: Nat Commun. 2025 May 31;16:5079. doi: 10.1038/s41467-025-60417-w (PMC12126495; doi:10.1038/s41467-025-60417-w)
Supplement: Supplementary file 2 — Description of Additional Supplementary Files [file 41467_2025_60417_MOESM2_ESM.docx]

Description of Supplementary Data files:

Supplementary Data 1: Eomes RIME Assay report results

Supplementary Data 2: Eomes RIME Pegasus T-test results

Supplementary Data 3: Gene Ontology full lists

Supplementary Data 4: Gene table list of Cut&Run-Seq, ATAC-Seq and RNA-Seq data
