# Supplementary material for: Eomesodermin in conjunction with the BAF complex promotes expansion and invasion of the trophectoderm lineage
Source: Nat Commun. 2025 May 31;16:5079. doi: 10.1038/s41467-025-60417-w (PMC12126495; doi:10.1038/s41467-025-60417-w)
Supplement: Supplementary file 7 — Reporting Summary [file 41467_2025_60417_MOESM7_ESM.pdf]

nature portfolio

Corresponding author(s): Elizabeth Robertson

Last updated by author(s): 26/02/2025

Reporting Summary

Nature Portfolio wishes to improve the reproducibility of the work that we publish. This form provides structure for consistency and transparency in reporting. For further information on Nature Portfolio policies, see our Editorial Policies and the Editorial Policy Checklist.

Statistics

For all statistical analyses, confirm that the following items are present in the figure legend, table legend, main text, or Methods section.

n/a	Confirmed	
☐	☒	The exact sample size (*n*) for each experimental group/condition, given as a discrete number and unit of measurement
☒	☐	A statement on whether measurements were taken from distinct samples or whether the same sample was measured repeatedly
☐	☒	The statistical test(s) used AND whether they are one- or two-sided<br>*Only common tests should be described solely by name; describe more complex techniques in the Methods section.*
☐	☒	A description of all covariates tested
☒	☐	A description of any assumptions or corrections, such as tests of normality and adjustment for multiple comparisons
☐	☒	A full description of the statistical parameters including central tendency (e.g. means) or other basic estimates (e.g. regression coefficient) AND variation (e.g. standard deviation) or associated estimates of uncertainty (e.g. confidence intervals)
☐	☒	For null hypothesis testing, the test statistic (e.g. *F*, *t*, *r*) with confidence intervals, effect sizes, degrees of freedom and *P* value noted<br>*Give P values as exact values whenever suitable.*
☒	☐	For Bayesian analysis, information on the choice of priors and Markov chain Monte Carlo settings
☒	☐	For hierarchical and complex designs, identification of the appropriate level for tests and full reporting of outcomes
☒	☐	Estimates of effect sizes (e.g. Cohen's *d*, Pearson's *r*), indicating how they were calculated

Our web collection on statistics for biologists contains articles on many of the points above.

Software and code

Policy information about availability of computer code

Data collection	The sequencing data has been deposited in NCBI GEO under the accession codes GSE276039, GSE276042 and GSE276261. Proteomic data has been deposited in the MassIVE repository, accessible via the link ftp://massive-ftp.ucsd.edu/v09/MSV000097887/
Data analysis	Statistical analysis performed using GraphPad Prism (10.0.2). ImageJ (2.14.0/1.54f) and Adobe Illustrator 2024 were used for image and figure preparation, respectively. All other programs used are indicated in the methods section.<br>PEAKS Studio version 8 built 20.<br>Bowtie2 version 2.4.4<br>SAMtools version 1.14<br>PICARD version 2.27.4<br>MACS2 version 2.2.7.1<br>deepTools version 3.5.1<br>Genomic Regions Enrichment Analysis Tool (GREAT) 4.0.4<br>SeqMonk 1.48.0

For manuscripts utilizing custom algorithms or software that are central to the research but not yet described in published literature, software must be made available to editors and reviewers. We strongly encourage code deposition in a community repository (e.g. GitHub). See the Nature Portfolio guidelines for submitting code & software for further information.

Data

Policy information about availability of data

All manuscripts must include a data availability statement. This statement should provide the following information, where applicable:

- Accession codes, unique identifiers, or web links for publicly available datasets
- A description of any restrictions on data availability
- For clinical datasets or third party data, please ensure that the statement adheres to our policy

The sequencing data has been deposited in the NCBI GEO database under accession code (GSE276039 (ATAC-seq), GSE276042 (CUT&RUN) and GSE276261 (RNA-seq). Proteomic data has been deposited in the MassIVE repository server, accessible via the link ftp://massive-ftp.ucsd.edu/v09/MSV000097887/. Supplementary Data1, 2, 3 & 4 contain further raw data of graphs included in the paper

Research involving human participants, their data, or biological material

Policy information about studies with human participants or human data. See also policy information about sex, gender (identity/presentation), and sexual orientation and race, ethnicity and racism.

Reporting on sex and gender	*Use the terms sex (biological attribute) and gender (shaped by social and cultural circumstances) carefully in order to avoid confusing both terms. Indicate if findings apply to only one sex or gender; describe whether sex and gender were considered in study design; whether sex and/or gender was determined based on self-reporting or assigned and methods used. Provide in the source data disaggregated sex and gender data, where this information has been collected, and if consent has been obtained for sharing of individual-level data; provide overall numbers in this Reporting Summary. Please state if this information has not been collected. Report sex- and gender-based analyses where performed, justify reasons for lack of sex- and gender-based analysis.*
Reporting on race, ethnicity, or other socially relevant groupings	*Please specify the socially constructed or socially relevant categorization variable(s) used in your manuscript and explain why they were used. Please note that such variables should not be used as proxies for other socially constructed/relevant variables (for example, race or ethnicity should not be used as a proxy for socioeconomic status). Provide clear definitions of the relevant terms used, how they were provided (by the participants/respondents, the researchers, or third parties), and the method(s) used to classify people into the different categories (e.g. self-report, census or administrative data, social media data, etc.) Please provide details about how you controlled for confounding variables in your analyses.*
Population characteristics	*Describe the covariate-relevant population characteristics of the human research participants (e.g. age, genotypic information, past and current diagnosis and treatment categories). If you filled out the behavioural & social sciences study design questions and have nothing to add here, write "See above."*
Recruitment	*Describe how participants were recruited. Outline any potential self-selection bias or other biases that may be present and how these are likely to impact results.*
Ethics oversight	*Identify the organization(s) that approved the study protocol.*

Note that full information on the approval of the study protocol must also be provided in the manuscript.

Field-specific reporting

Please select the one below that is the best fit for your research. If you are not sure, read the appropriate sections before making your selection.

[x] Life sciences [ ] Behavioural & social sciences [ ] Ecological, evolutionary & environmental sciences

For a reference copy of the document with all sections, see nature.com/documents/nr-reporting-summary-flat.pdf

Life sciences study design

All studies must disclose on these points even when the disclosure is negative.

Sample size	Experiments were performed with a minimum of n=3 independent biological samples. Sample sizes were selected based on previous experiments
Data exclusions	No data excluded
Replication	Replicate experiments were successfully validated
Randomization	No randomization performed. To control for effects of covariates, we used wildtype littermates or untreated and control vehicle treatment
Blinding	As the phenotype of the control versus the Eomes null embryos was morphologically evident, blinding was not required

Reporting for specific materials, systems and methods

We require information from authors about some types of materials, experimental systems and methods used in many studies. Here, indicate whether each material, system or method listed is relevant to your study. If you are not sure if a list item applies to your research, read the appropriate section before selecting a response.

Materials & experimental systems

n/a	Involved in the study
☐	☒ Antibodies
☐	☒ Eukaryotic cell lines
☒	☐ Palaeontology and archaeology
☐	☒ Animals and other organisms
☒	☐ Clinical data
☒	☐ Dual use research of concern
☒	☐ Plants

Methods

n/a	Involved in the study
☒	☐ ChIP-seq
☒	☐ Flow cytometry
☒	☐ MRI-based neuroimaging

Antibodies

Antibodies used

Antibody information is provided in Supplemental Table 1
Rat monoclonal IgG2a anti-Eomes eBioscience 14-4875-82 Lot no. 2493129 1:500 Immunofluorescence (IF)
Rabbit polyclonal anti-AP-2γ Cell Signaling 2320S Lot no. 3 1:100 IF
Rabbit monoclonal IgG anti-FGFR1 Cell Signaling 9740S Lot no. 4 1:100 IF
Phalloidin AF633 ThermoFisher A22284 1:100 IF
Polyclonal goat anti-HAND1 R&D AF3168 Lot no. WTD0423011 1:100 IF
Rabbit polyclonal anti-RFP Rockland 600-401-379 Lot no. 48710 1:200 IF
Donkey anti-rat AF488 Invitrogen A-21208 Lot no. 2482958 1:400 IF
Donkey anti-rat AF594 Invitrogen A-21209 Lot no. 2078918 1:400 IF
Donkey anti-rabbit AF488 Invitrogen A-21206 Lot no. 1927937 1:400 IF
Donkey anti-goat AF594 Invitrogen A-11058 Lot no. 2445414 1:400 IF
Donkey anti-rabbit AF594 Invitrogen A-21207 Lot no. 2313074 1:400 IF
Rat monoclonal IgG2a anti-Eomes eBioscience 14-4875-82 Lot no. 2493129 1:10,000 Western Blot (WB)
Rabbit polyclonal anti-β-tubulin Cell Signaling 2146S Lot no. 10 1:2,000 WB
Goat anti-rat IgG, HRP conjugated Cell Signaling 7077S Lot no. 14 1:2,000 WB
Donkey anti-rabbit IgG, HRP conjugated Amersham NA934V Lot no. 17271476 1:2,000 WB
Guinea Pig anti-Rabbit IgG (Heavy & Light Chain) Antibodies online ABIN101961 Lot no. NE-200-022001 1:100 CUT&RUN
Rabbit polyclonal anti-Eomes abcam ab23345 Lot no. GR3304549-1 1:50 CUT&RUN
Rabbit polyclonal anti-Eomes abcam ab23345 Lot no. GR3390346-2 RIME

Validation

Validation information has been taken from the manufacturer's website for the primary and secondary antibodies listed in Supplemental Table 1

Eukaryotic cell lines

Policy information about cell lines and Sex and Gender in Research

Cell line source(s)	Eomes-degron trophoblast stem cells, as produced in this study
Authentication	By the Robertson lab in this study
Mycoplasma contamination	None
Commonly misidentified lines (See ICLAC register)	*Name any commonly misidentified cell lines used in the study and provide a rationale for their use.*

Animals and other research organisms

Policy information about studies involving animals; ARRIVE guidelines recommended for reporting animal research, and Sex and Gender in Research

Laboratory animals	Eomes null (delta 2-5) crossed with an Rosa26-MT/MG allele, as stated in the methods section
Wild animals	n/a

Reporting on sex	n/a as peri-implantation embryos were studied
Field-collected samples	n/a
Ethics oversight	All animal procedures were performed in accordance with the Home Office (UK) guidelines and authorized by the local Animal Welfare and Ethical Committee.

Note that full information on the approval of the study protocol must also be provided in the manuscript.

Plants

Seed stocks	*Report on the source of all seed stocks or other plant material used. If applicable, state the seed stock centre and catalogue number. If plant specimens were collected from the field, describe the collection location, date and sampling procedures.*
Novel plant genotypes	*Describe the methods by which all novel plant genotypes were produced. This includes those generated by transgenic approaches, gene editing, chemical/radiation-based mutagenesis and hybridization. For transgenic lines, describe the transformation method, the number of independent lines analyzed and the generation upon which experiments were performed. For gene-edited lines, describe the editor used, the endogenous sequence targeted for editing, the targeting guide RNA sequence (if applicable) and how the editor was applied.*
Authentication	*Describe any authentication procedures for each seed stock used or novel genotype generated. Describe any experiments used to assess the effect of a mutation and, where applicable, how potential secondary effects (e.g. second site T-DNA insertions, mosiacism, off-target gene editing) were examined.*
